# Mutation on *lysX* from *Mycobacterium avium hominissuis* impacts the host–pathogen interaction and virulence phenotype

**DOI:** 10.1080/21505594.2020.1713690

**Published:** 2020-01-29

**Authors:** Greana Kirubakar, Hubert Schäfer, Volker Rickerts, Carsten Schwarz, Astrid Lewin

**Affiliations:** a Division 16, Mycotic and Parasitic Agents and Mycobacteria, Robert Koch Institute, Berlin, Germany; b Pediatric Pneumology, Immunology and Intensive Care Medicine, Division of Cystic Fibrosis, Charité - Universitätsmedizin Berlin, Berlin, Germany

**Keywords:** Virulence, *Mycobacterium avium hominissuis*, nontuberculous mycobacteria, Galleria mellonella infection model, multinucleated giant cells, pathogenesis, *lysX* (lysyl-tRNA synthetase), host-pathogen interaction, inflammatory cytokines

## Abstract

The *lysX* gene from *Mycobacterium avium hominissuis* (MAH) is not only involved in cationic antimicrobial resistance but also regulates metabolic activity. An MAH *lysX* deficient mutant was shown to exhibit a metabolic shift at the extracellular state preadapting the bacteria to the conditions inside host-cells. It further showed stronger growth in human monocytes. In the present study, the LysX activity on host–pathogen interactions were analyzed. The lysX mutant from MAH proved to be more sensitive toward host-mediated stresses such as reactive oxygen species. Further, the lysX mutant exhibited increased inflammatory response in PBMC and multinucleated giant cell (MGC) formation in human macrophages during infection studies. Coincidentally, the lysX mutant strain revealed to be more reproductive in the *Galleria mellonella* infection model. Together, these data demonstrate that LysX plays a role in regulating the bacillary load in host organisms and the lack of *lysX* gene facilitates MAH adaptation to intracellular host-habitat, thereby suggesting an essential role of LysX in the modulation of host–pathogen interaction.

## Introduction

Humans come in contact with different mycobacterial species as these are omnipresent in diverse environmental niches [1]. Their competency to multiple adaptations enables many mycobacterial species to inhabit a vast array of environmental habitats, including natural waters, soils, sawdust, and drinking water distribution systems [2]. One mechanism mediating adaptation is the presence of a lipid-rich hydrophobic outer membrane, which is a major determinant of surface adherence, biofilm formation, aerosolization, and antibiotic/disinfectant resistance [2,3].

Phylogenetically, the genus *Mycobacterium* is classified into three main groups: (i) obligate pathogens that require a mammalian host such as *Mycobacterium* (*M*.) *tuberculosis* (MTB) complex; (ii) opportunistic environmental mycobacteria referred to as Nontuberculous mycobacteria (NTM) and (iii) species that thrive in a “strictly intracellular” niche such as the *Mycobacterium leprae* [4]. The NTM can be further divided into slow growers (e.g. *M. avium* complex (MAC)) and rapid growers (e.g. *M. abscessus*).

Apart from *M. tuberculosis* (MTB) and *M. leprae*, the slow-growing NTM are also frequently involved in human disease [5,6]. Amongst them, the *M. avium* subsp. *hominissuis* (MAH) poses a major risk to immunocompromised patients, especially those who are infected with HIV and have severe respiratory diseases such as cystic fibrosis, bronchiectasis, and chronic obstructive pulmonary disease (COPD) [7]. Subacute or chronic lymphadenitis in the cervical region are the most common manifestation of MAH in children [8]. MAC also causes lung disease very similar to tuberculosis, manifesting as apical fibrocavitary disease or interstitial nodular infiltrates in immunocompetent hosts and bronchiectasis [9]. In this manner, the clinical symptoms of pulmonary infection by NTM are similar to other pulmonary diseases, consequently the NTM diseases are likely misdiagnosed and underreported especially in developing countries, where TB and AIDS are the major focus of the healthcare system [10]. On account of the numerous types of NTMs being typically resistant to antituberculosis drugs [5], it is convincing that they are being misinterpreted as multidrug-resistant (MDR) MTB strains [11]. Recently it was also reported that among the chronic TB and MDR-TB patients from developing countries, 12–30% of them were found to suffer from NTM infections [12]. This eventually leads to inaccurate diagnosis contributing to disease progression and deterioration of the patient’s health.

On the other hand, the incidence of NTM lung diseases has also been increasing worldwide over the past few decades [13] posing it to be a pathogen of a global health threat. It was reported that NTM diseases lead to a greater disease burden than TB in the US, Canada, Japan, Korea, Australia, and the United Kingdom [14–18]. For example, in England and Ireland, the annual incidence rates of NTM diseases was increased from 5.6/100,000 in the year of 2007 to 7.6/100,000 in 2012 [18]. Despite this, mechanisms of pathogenicity for the heterogeneous group of NTM remain scarce. Hence, there exists an urgency to discover the virulence mechanisms of these environmental pathogens in order to improve the accuracy in diagnosis and relevancy in the treatment process. Although several studies have exposed some knowledge on the *M. avium* genes associated with pathogenesis, a deeper understanding is still required about the mycobacterial virulence determinants [19].

The multidrug resistance and the virulent nature of the environmental pathogen *Mycobacterium avium* is ascribed to the organism’s lipid-rich cell wall and its colony morphology [20]. Several cell-wall associated lipids are considered to be potential mediators of virulence. For instance, *M. avium* expresses on its cell surface serovar-specific glycopeptidolipids (ssGPLs) [21] which are absent in MTB. These highly antigenic GPLs have been involved in inhibiting macrophage activation [22,23] and in immuno-modulatory activity by downregulating Th_1_ type responses, thus benefiting the pathogen’s intracellular survival [24,25]. In addition, by changing the pattern of GPL expression *M. avium* can also modulate macrophage inflammatory responses [24].

Studies on the *lysX* gene from MTB revealed that the gene which is involved in the lysinylation of surface phospholipids was responsible for the susceptibility toward antimicrobial peptides in-vitro and for the survival of the pathogen in vivo [26]. These findings suggest an association between the susceptibility of pathogenic mycobacteria to antimicrobial peptides and the components on the bacterial surface, whose expression may also vary depending on the environment [27].

Unlike MTB, the *M. avium* survive in harsh environments including environmental hosts like free-living amoebae and thus have multifarious survival mechanisms [7]. Additionally, it is also assumed to have a cell wall which is harder to penetrate distinguishing *M. avium* from MTB [27]. Thus, it is of key importance to understand the interactions between the human host and *M. avium*, and decode how the *M. avium* are capable to survive host defense mechanisms. In our earlier experiments, we had already evidenced that the gene *lysX*, which is a lysyl-transferase-lysyl-tRNA synthetase from MAH is associated with resistance toward cationic antimicrobials as well as having an immense impact on the carbohydrate and lipid metabolism. Lack of *lysX* in MAH caused a shift in metabolism, which reflected the intracellular metabolic pattern of MTB amid infection, thereby improving the ability to multiply within human monocyte-derived cells. Notably, this behavior was contrasting to that of the lysX mutant from MTB [28]. In this manuscript, we have attempted to analyze the effect of the MAH *lysX* gene in host–pathogen interactions. Interestingly, the lysX mutant strain displayed increased reproducibility in human monocytes and galleria larvae even though the mutant remained sensitive to host-mediated stresses at the extracellular level.

## Materials and methods

### Bacterial strains and growth conditions

The study was performed with the wild-type strain MAH 104 [29], the LysX-deficient mutant lysXmut and the complemented strain lysXcomp [28,30]. Middlebrook 7H9 broth (BD Biosciences) along with 0.05% Tween 80 was used to grow the mycobacterial strains at 37°C and the media was supplemented with 10%-modified ADC (2% glucose, 5% BSA, 0·85% NaCl). The strains were cultured without shaking at 37°C. Middlebrook 7H11 agar plates supplemented with 10% modified ADC or OADC (oleic acid, albumin, dextrose, catalase; BD Biosciences) and 0.5% Glycerol was also used for plating purposes.

#### Stress resistance test

### H_2_O_2_ and NO exposure

The bacterial cultures were grown up to an OD of 1.8 and then were exposed to a single dose of H_2_O_2_ or the NO donor DETA/NO (diethylenetriamine/nitric oxide adduct). Doses of 20 mM and 100 mM H_2_O_2_ or 25 mM DETA/NO were used. The ATP production of each of the cultures was monitored after 4 h post addition of stress. The ATP content was quantified using the luminescence-based kit BacTiter-Glo™ Microbial Cell Viability Assay (Promega) as per the manufacturer’s instructions. A microplate luminometer, TriStar LB 941 Multimode Microplate Reader (Berthold Technologies) was used to measure the luminescence as relative light units (RLU). All stress resistance tests were performed in triplicate and repeated independently three times.

### Exposure to human beta defensin-1

The activity of human beta defensin-1 against MAH was determined by a 96-well microplate assay. 160 µl of dilutions of human beta defensin-1 (Sigma-Aldrich) and 40 μl log-phase bacteria (OD of 1.8) were added to the microplate wells to obtain a final concentration of 0.5 µg/ml or 1 μg/ml of peptide. The defensin was dissolved in 0.01% acetic acid and 0.01% acetic acid was added to the controls. After the exposition for 5 days at 37°C, ATP content was measured as aforesaid to check the growth inhibitory activity of the peptide. The experiments were performed in triplicate each being repeated three times.

#### Quantification of cytokines produced by infected human PBMCs

Human buffy coats were utilized to extract peripheral blood mononuclear cells (PBMCs) by Ficoll-Paque (GE Healthcare) gradient centrifugation as mentioned by the manufacturer’s recommendations and method as described elsewhere [31]. PBMCs were seeded in 24-well cell culture plates (TPP) at 10^6^ cells/well and incubated at 5% CO_2_, 37°C for 24 h prior to stimulation with bacteria. The PBMCs were infected with the bacterial strains at an MOI of 10 and were incubated for up to 120 h at 5% CO_2_ at 37°C. Uninfected cells were used as negative controls. The culture supernatants were collected at 24 h and 120 h post infection and analyzed for cytokine secretion by ELISA (Human IL-10, IL-1β, IL-12(P40) & TNFα, ELISA Ready-SET-Go!™, Thermo Fischer Scientific), in accordance with the manufacturer’s instructions. Cytokine quantifications were repeated with three buffy coat PBMCs from different donors including three replicates per repeat.

### Glycopeptidolipid (GPL) extraction

GPLs were prepared as in accordance with methods as described in [32,33]. The *M. avium* strains were cultivated in Middlebrook 7H9 broth with 0.05% Tween 80 (Becton Dickinson) supplemented with modified ADC enrichment at 37°C. The bacterial strains were grown up to a log phase of OD_600_ 1.8–2.0, were heat-killed and the GPL was extracted from the dried bacterial pellets. In brief, the dried pellets (from 50 ml culture) were extracted with 10 ml of chloroform-methanol (2:1, vol/vol) involving ultrasonication (Branson sonifier-450 D, G. Heinemann) at 100% power for 1 min. The liquid phases were then hydrolyzed with 3 ml of 0.2 N sodium hydroxide in methanol to remove alkali-labile lipids. The lipid extracts were then neutralized to pH 7 using 6 N HCl. Chloroform (6 ml) was then added, shaken well following the addition of water (5 ml) and centrifuged at 8000 g for 10 min. The bottom phase containing the organic solvent was pipetted into a new tube and then evaporated completely. The alkali-stable lipids were dissolved in chloroform-methanol (2:1) and applied to silica-coated TLC plates (Analtech). The GPLs were separated by thin-layer chromatography (TLC) employing the solvent system of chloroform/methanol (90:10, vol/vol). The GPLs were displayed by spraying the plates with 10% H_2_SO_4_ in ethanol, followed by the exposition of the plate to hot air.

### GPL antigen – ELISA

The GPLs extracted from the bacterial strains were diluted in PBS (1:1000) and 100 µl of the diluted GPLs were added to a polystyrene microplate well (Nunc MaxiSorp, Thermo Fischer Scientific). After that, the plates were sealed and incubated overnight at 4°C. Nonspecific binding was blocked using 3% BSA dissolved in PBS and then the wells were washed with PBS. Serum from patients with or without MAH infection (approval from the ethics committee of the Charité – Universitätsmedizin Berlin (EA2/093/12)) was diluted (1:1000) using 1% BSA dissolved in PBS and 100 µl were applied to the appropriate wells which contained the GPL extracts and incubated at room temperature for an hour. GPLs without the exposure to serum were used as a negative control. Then, the plate was washed with PBS-Tween 20 and the wells were treated with 100 µl of horseradish peroxidase (HRP)-goat anti-human IgG (diluted to 1:5000 in 1% BSA dissolved in PBS) as the secondary antibody. After incubation for an hour, TMB ELISA substrate (Sera care) was added and the reaction was stopped after 10 min by adding 0.16 M H_2_SO_4_. The absorption was determined using a microplate reader (Infinite M200 PRO, Tecan) at 450 nm. All assays were performed in triplicates and repeated with GPL preparations from three independent cultures of each strain.

### Measurement of fusion rates of infected human monocyte-derived macrophages

The induction of the fusion of human monocyte-derived macrophages (HMDM) upon infection with mycobacteria has been described previously [34]. The human monocytes were pre-activated with 100 U/mL IFNγ and then infected at an MOI of 10. The infection experiment was performed in imaging cell culture dishes (ibidi GmbH) consisting of labeled grids. The ibidi dishes were employed as they facilitate good optical quality for high-resolution microscopy and the grids aid in the quantification of multinucleated giant cells (MGCs).

After 5-days postinfection, the culture samples were fixed with 4% paraformaldehyde and then stained with Nile red (25 mg in 2.5 ml of dimethyl sulfoxide) which stains neutral lipids and DAPI (5 mg/ml (stock), 1:100 diluted in dimethyl sulfoxide) which stains DNA. The stained preparation was evaluated using confocal laser scanning microscopy (LSM780; Carl Zeiss). To determine the fusion index the numbers of nuclei per macrophage were counted. Macrophages containing at least three nuclei were considered as multi-nucleated and the number of nuclei present in multi-nucleated cells was also determined. At least 500 nuclei were counted per preparation and the fusion index (FI) was calculated using the formula:ref [34].

$$FI\%=\;\frac{Number\;of\;nuclei\;in\;multinucleated\;cell}{Total\;number\;of\;nuclei}\times \;100$$

The experiment was repeated five times with HMDM from five buffy coats (different donors) including three replicates each repeat.

#### Galleria mellonella *infection*

*Galleria mellonella* (Greater wax moth) larvae were received from the animal breeding facility at the Robert Koch Institute, Berlin, Germany. Larvae were kept at 27°C. Healthy larvae were identified by their cream color, without dark discoloration and larvae with a bodyweight of approximately 250 mg were used in infection experiments. For inoculation, the larvae were injected by a 30G insulin syringe needle (Omnican, Braun) in the hind leg with 10^6^ bacteria in a volume of 20 μl (n = 30 per group). After inoculation, the infected larvae were kept individually (one larvae per well) in 12-well cell culture plates in an alternate manner and then were incubated at 37°C. The wells were equipped with woodchips. Untouched larvae and PBS injected worms served as control groups. The larvae were checked every day for survival, pupation transitions and death events were recorded. The survival curve was performed in Graph Pad Prism.

Apart from quantifying the survival of infected *Galleria* larvae we also quantified the survival of the MAH strains within *Galleria*. Three to five living larvae were used for homogenization and were freeze-killed at −20°C immediately after inoculation to assess for uniform inoculation as well as actual bacterial infection rate, and an equal number of larvae per group were sacrificed after 5 and 10 days postinfection. Non-melanized larvae were selected in order to obtain a uniform test group. The sacrificed larvae were disinfected by wiping down with 70% ethanol and homogenized with glass beads (0.11 mm diameter; Sartorius) in PBS-Tween 80 using a Precellys-24 tissue homogenizer (Peqlab). CFU evaluation was carried out by plating serial dilutions of the homogenized lysates in duplicate on to Middlebrook 7H11 agar plates containing cycloheximide (25 µg/ml) and Vancomycin (2 μg/ml) (Sigma) to inhibit the growth of intrinsic *G. mellonella* flora. The mean CFU/ml of MAH surviving in *G. mellonella* was determined after 0, 5 and 10 days postinfection. In total, 150 larvae (30 per group) were used per experimental trial and infection experiments were repeated on three independent occasions.

### Image processing

Images were adjusted for the appropriate dimensions, for optimal brightness and contrast (applied to the whole image), employing Photoshop Lightroom (Adobe Systems).

#### Statistical analysis

All values mentioned in the results section are the mean of at least three independent experimental repeats ± SD. At least three technical replicates per repeat were done. Experimental data were tested for statistical significance at p < 0.05 using a Student’s t-test (Microsoft Excel 2010) and graphically represented using GraphPad Prism. The unpaired two-tailed Student’s t-tests are reported for pair-wise statistics between the strains (lysX mutant vs wild type and lysX mutant vs lysX complement) which is provided in figure legends.

## Results

### LysX influences tolerance toward oxidative and nitrosative stress

**Figure 1.**
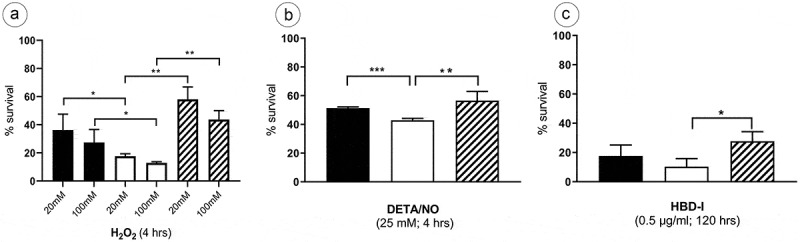
Sensitivity of MAH strains to hydrogen peroxide, nitric oxide, and defensin. Survival of *M. avium hominissuis* wild type (MAH 104) (black bar), lysX mutant (open bar) and lysX complemented strain (striped bar) bacteria after incubation with (a) 20 mM and 100 mM H_2_O_2_ for 4 h (b) 25 mM DETA/NO for 4 h or (c) 0.5 µg/ml Human Beta Defensin-1 for 5 days determined by measurement of ATP production using an luminescence-based assay. Data were normalized to untreated controls (100% survival). Data are means ± standard deviation of the results from three independent experiments, each performed in triplicate (with three cultures). Statistically significant differences in percentage survival relative to that of lysX mutant strain are indicated by asterisks (*, P < 0.05; **, P < 0.01; ***, P < 0.0001; two-tailed, unpaired Student’s t-test)

The effect of macrophage‐mediated host defense mechanisms such as reactive oxygen intermediates (ROI) and reactive nitrogen intermediates (RNI) against MAH was examined. The wild type, lysX mutant and the lysX complemented strains, growing in a mid-logarithmic phase were exposed to H_2_O_2_ concentrations of 20 mM and 100 mM. The ATP content of the cultures was determined after 4 h of incubation using the BacTiter-Glo™ Microbial Cell Viability Assay with RLU (Relative Light Units) as readout. Exposure to both the H_2_O_2_ concentrations resulted in a considerable reduction in the cell viability of the lysX mutant strain in comparison to the other strains. While, for example, the wild type was reduced from 100% RLU if not treated with H_2_O_2_ to 36% if treated with 20 mM H_2_O_2_ for 4 h, the mutant was reduced to 17% by the same stress condition (Figure 1(a)). The bacterial strains were also exposed to 25 mM NO donor (DETA/NO) for a period of 4 h. Although with a decrease to 51% in the wild type versus 42% in the mutant the effect was less pronounced as for oxidative stress, the lysX mutant strain again proved to be slightly more sensitive toward the NO treatment (Figure 1(b)).

### Absence of lysX slightly increases the susceptibility to HBD-1 defensin

The antimycobacterial activity of the Human Beta Defensin–I (HBD-I) belonging to the cationic antimicrobial peptide family against the three MAH strains was examined. All strains showed a strong reduction in cell viability after an exposure to 0.5 µg/ml of HBD-1 for 120 h. Compared to untreated cells (100% RLU) the wild-type MAH 104 only showed a value of 17% after exposure to HBD-I, the mutant was reduced to 10% and the complemented strain to 21% (Figure 1(c)). Thus, the *lysX* mutation had slightly increased the defensin susceptibility of MAH 104.

### The inflammatory and anti-inflammatory response of MAH-infected human PBMCs is influenced by lysX

**Figure 2.**
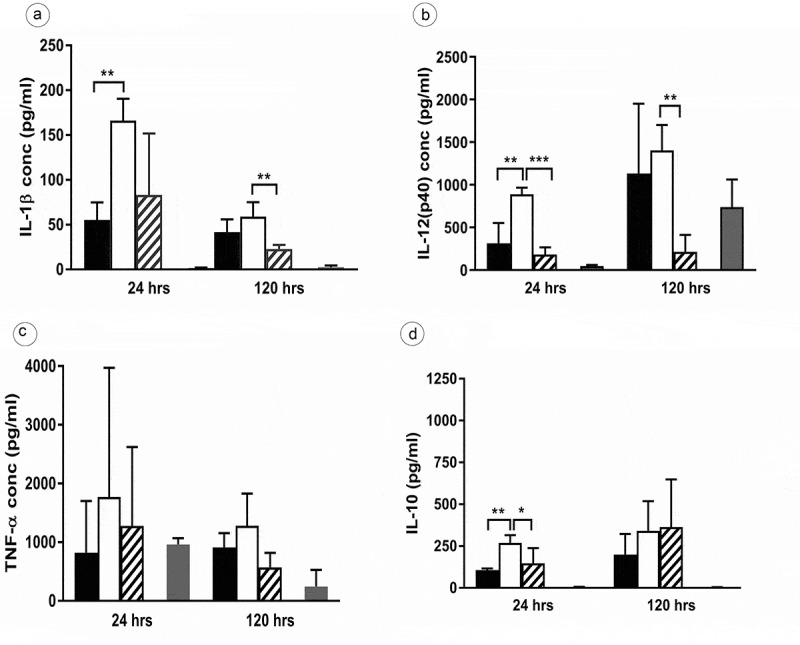
Inflammatory cytokine secretions of human PBMC infected with *M.* *avium* strains. Human blood-derived PBMCs were infected with the MAH strains (MOI 10) wild type (Black bar), lysX mutant (Open bar), lysX complemented strain (striped bar) and pro-inflammatory (IL-1β (a), IL- 12 (b), TNF-α (c) and anti-inflammatory (IL-10 (d)) cytokines release was measured by ELISA using the kits (ELISA Ready-SET-Go!™ Kit Thermo Fischer Scientific). Untreated cells (gray) were used as controls. Three independent experiments were done in triplicates, and data shown are the mean with the standard deviation of the three experiments. Statistically significant differences relative to that of lysX mutant strain are indicated by asterisks (*, P < 0.05; **, P < 0.01; ***, P < 0.0001; two-tailed, unpaired Student’s t-test).

In previous studies, we reported that the MAH lysX mutant displayed an enhanced intracellular growth in human blood-derived monocytes [28]. The observed phenotypic trait may be affected by a differential host inflammatory response. In order to analyze cytokine production by PBMCs stimulated with the wild type, the mutant, and the complemented strains (MOI 10), the cell supernatants were harvested at 24 h and 120 h (120 h as the time point wherein the lysX mutant showed an elevated intracellular growth in monocytes [28]) after infection for quantification of the pro-inflammatory cytokines IL-1β, IL-12(p40) and TNF-α as well as the anti-inflammatory cytokine IL-10. Comparatively, the lysX mutant strain induced more pro-inflammatory and anti-inflammatory cytokine secretion than the wild type at both the time points of infection (Figure 2). Statistically significant differences were observed for IL-1β, IL-12(P40) and IL-10, while the differences for TNF-α were not statistically significant. The differences in induction of cytokine secretion between wild type and mutant were more pronounced after 24 h compared to 120 h infection time.

### LysX interferes with GPL expression and its antigenic reactivity

**Figure 3.**
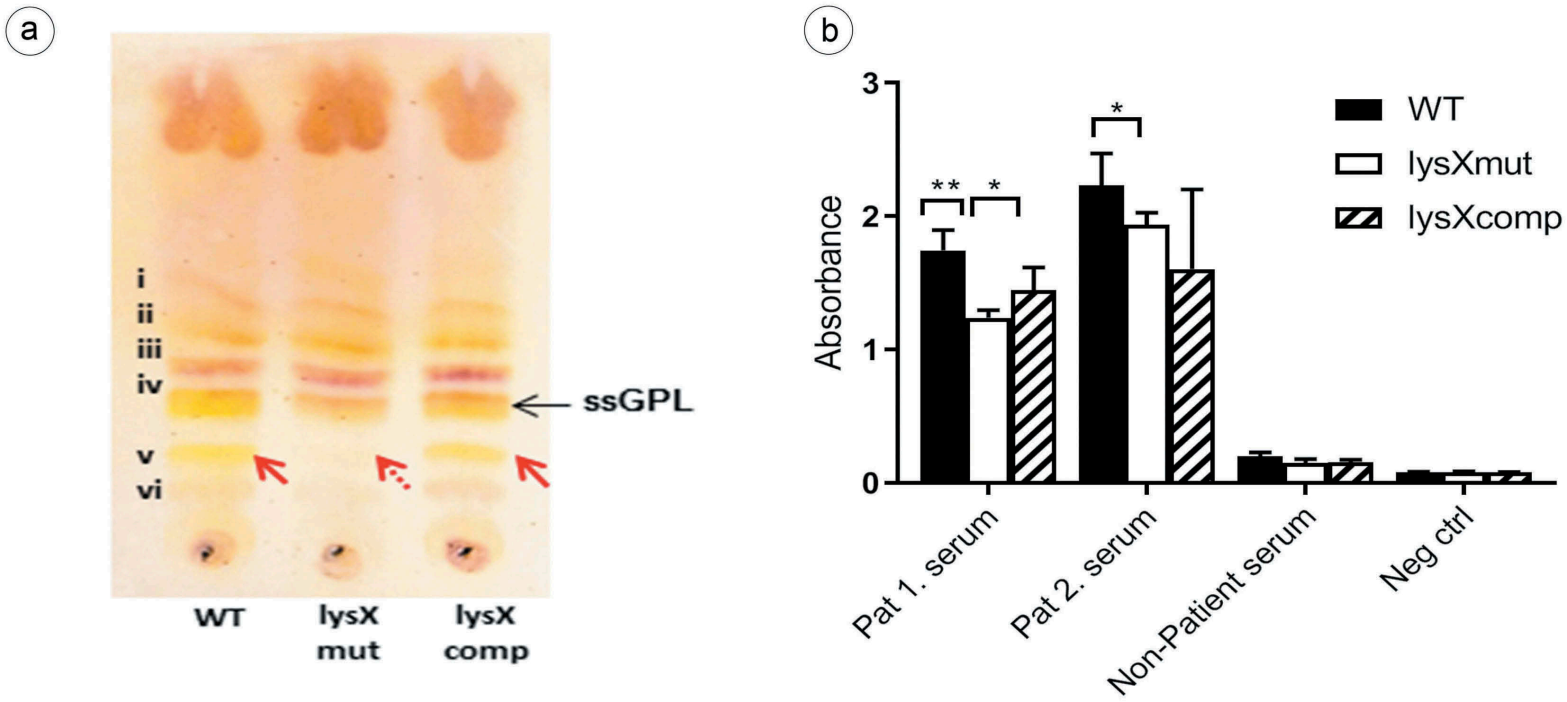
GPL expression and immune reactivity of *M. avium* strains. (a) Thin-layer chromatography (TLC) of GPL extracted from MAH strains wild type (Lane 1); lysX mutant (Lane 2) and lysX complemented strain (Lane 3). Wild-type strain and lysX complemented strain both expressed similar GPL pattern whereas lysX mutant did not express one GPL band (band v) at lower mobility position (indicated with dashed-red arrow). Serovar‐specific (ss) GPLs (band iv) is marked with black arrow [37]. (b) ELISA showing reactivity of sera from two MAH-infected patients against MAH GPL extracts from strains wild type (WT), lysX mutant (lysXmut) and lysX complement (lysXcomp). GPL from lysXmut was less reactive to the MAC patient sera. Serum from a healthy person was included as a control. Three independent experiments were done and repeated with GPL preparations from three independently grown cultures per strain and the mean values are represented in the graph. Statistically significant differences relative to that of lysX mutant strain are indicated by asterisks (*, P < 0.05; **, P < 0.01; two-tailed, unpaired Student’s t-test).

GPLs are unique cell-wall molecules found in MAC species and modulate the release of various proinflammatory mediators, such as prostaglandins, leukotrienes, IL-1, IL-6, and TNF-α [25,35]. The ability of ssGPLs to stimulate the release of proinflammatory mediators appears to be structure-specific [35], and slight structural modifications can alter the way in which the GPL interacts with host-cell receptors [36]. Since the *lysX* gene is also associated with the membrane phospholipid composition, the expression of GPL in the MAH strains was also analyzed in this study. The effect of the *lysX* mutation on GPL production was investigated by analyzing purified alkali-stable lipids from bacterial cell walls. The GPL expression pattern was visualized by TLC. Representative results from the TLC analysis are shown in Figure 3(a). The analysis of lipid extracts from the wild type, mutant and complemented strains revealed a different expression profile of GPL in the mutant strain. The GPL bands were numbered (i to vi) based on relative mobility on the TLC plate, with the band (i) being the least polar (highest mobility on the TLC plate). The bands (i-iii) appeared to be apparently similar in all the three strains, but the band (v), a single GPL band, with a lower TLC mobility seemed to be lacking in the lysX mutant. The strain-specific GPL band (iv) was also identified [37].

It has been depicted that the anti-GPL antibody levels mirror the disease activity in MAC lung infections [38]. Therefore, the reactivity of sera from MAH-infected patients with Cystic Fibrosis against the GPL extracts from the MAH strains were tested by ELISA. As shown in Figure 3(b), MAC patient sera showed a higher mean absorbance value against GPLs extracted from the wild-type (1.989) when compared to the lysX mutant strain (1.588). The reactivity of the serum from a healthy person was only slightly above the negative control.

### LysX impacts the rate of macrophage fusion

MGCs are formed through the fusion of multiple macrophages during chronic antigenic stimulation, generated by virulent mycobacterial infections [39]. Since the *lysX* mutation was found to boost mycobacterial growth in monocytes [28], we tried to analyze the impact of the *lysX* mutation on fusion events.

**Figure 4.**
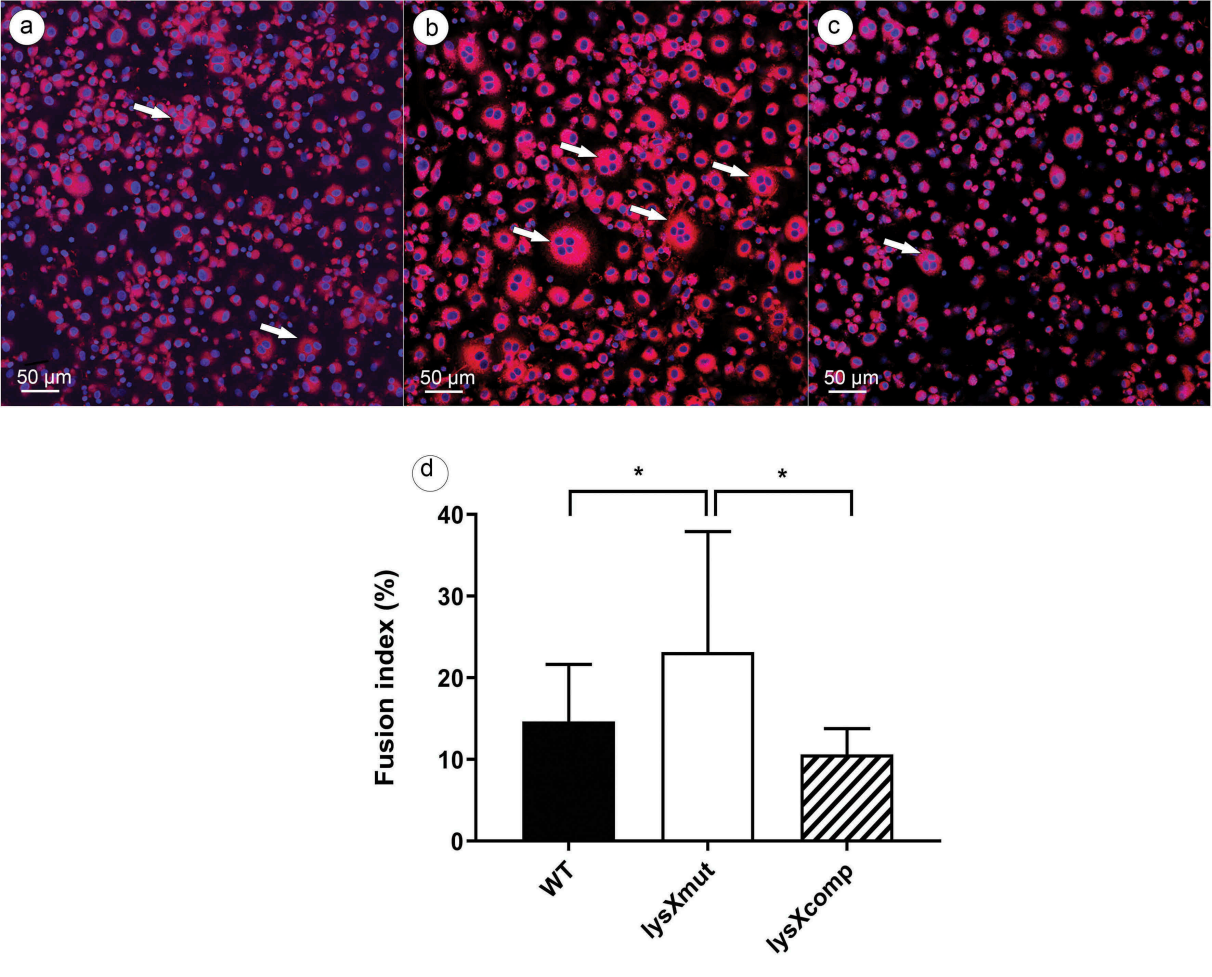
Formation of multi-nucleated cells after infection of human blood-derived monocytes with *M. avium* strains. Microscopic examination of human blood-derived monocytes infected (MOI 10) with *M. avium* strains (a) wild type (WT), (b) lysX mutant (lysXmut) and (c) lysX complemented strain (lysXcomp) at 5 days postinfection. Fluorescence microscopic images showing multinucleated giant cells (indicated with white arrows) generated by 4 million infected monocytes per culture dish. The cells were stained with Nile red and DAPI, red fluorescence showing the Nile red staining of neutral lipids and blue fluorescence showing the DAPI staining of the macrophage DNA. The stained samples were picturized using a confocal laser scanning microscope at a magnification of 100 × . Scale bars: 50 µM. (d) At least 500 nuclei were counted per sample to calculate the fusion index using the formula below [34]: $FI\%=\;\frac{Number\;of\;nuclei\;in\;multinucleated\;cell}{Total\;number\;of\;nuclei}\times \;100$ Data are means ± standard deviation of the results from five independent experiments using buffy coats from five different donors. Statistically significant differences relative to that of lysX mutant strain are indicated by asterisks (*, P < 0.05; two-tailed, unpaired Student’s t-test).

The HBDM were infected with bacterial strains at an MOI of 10 and samples were fixed at the fifth day postinfection for determination of the fusion index (FI). As shown in (Figure 4(a–d)), infection with the mutant caused a FI of 23% while the wild type and the complemented strain displayed a FI of 14% and 9%, respectively. The fusion indexes of the uninfected controls were always below 5%. Thus, the macrophages infected with the lysX mutant strain induced more MGC formation than the other two strains.

### LysX affects the virulence of MAH

*Galleria mellonella* larvae were used as an *in vivo* model to assess the difference in virulence potency of the three MAH strains. Both, the survival of infected larvae as well as the growth of MAH within the larvae were investigated.

**Figure 5.**
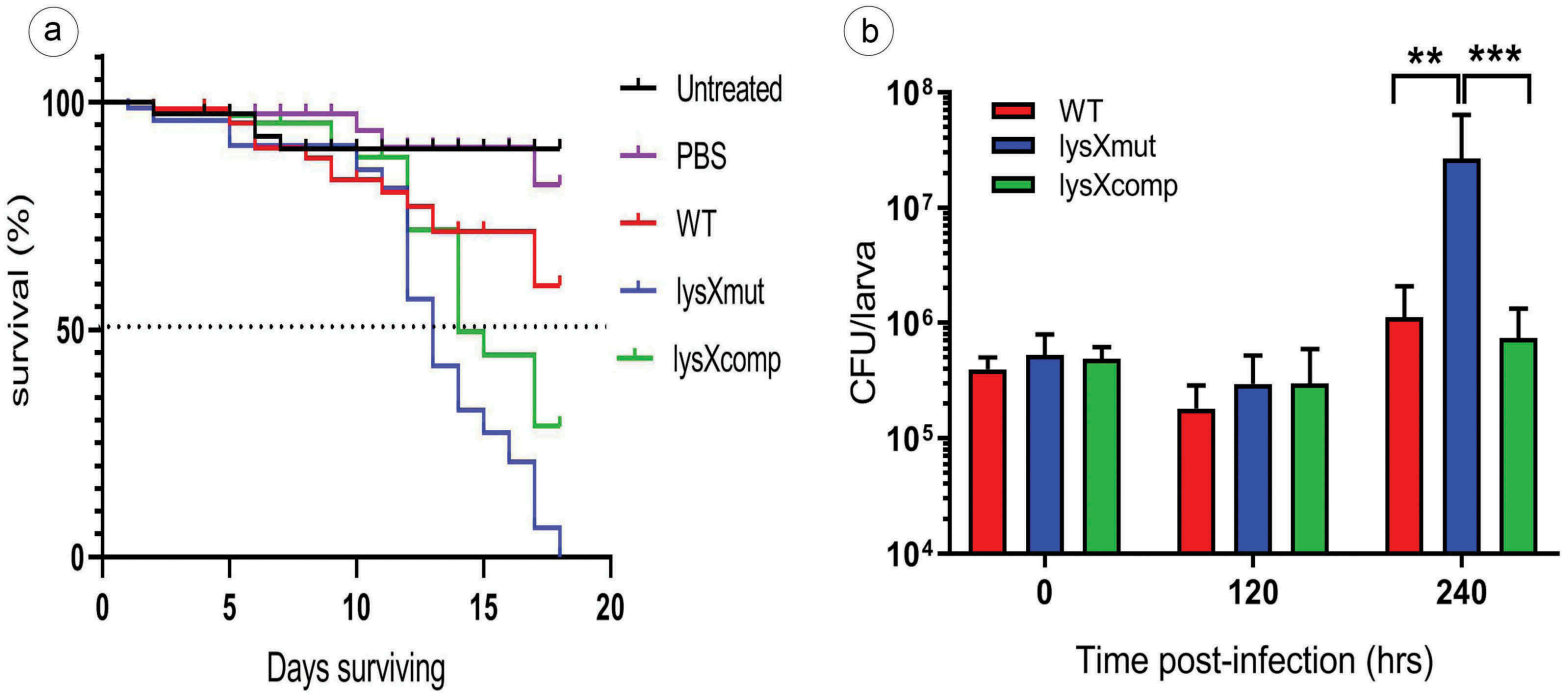
Virulence of MAH in *G. mellonella* larvae. (a) Survival of larvae infected with MAH. Larvae were infected with 10^6^ CFU of the MAH strains wild type (WT), lysX mutant (lysXmut) or lysX complemented strain (lysXcomp) on day 0. Controls were injected with PBS or were left untreated. Three different experiments using in each experiment 30 larvae per group were performed. The survival and pupation of the larvae were checked every day. Pupation events are indicated by short dashes. The graph shows the pooled survival percentages of the three experiments. (b) Quantification of bacterial growth *in vivo*: 3 to 5 larvae per group were sacrificed, homogenized, and plated for CFU counts on days 0, 5 and 10, respectively. The data represented are means ± standard deviation of the results from three independent experiments. Statistically significant differences relative to that of lysX mutant strain are indicated by asterisks (*, P < 0.05; **, P < 0.01; ***, P < 0.0001; two-tailed, unpaired Student’s t-test)

Larvae survival (n = 30 per group), in response to an inoculum of MAH of 10^6^ CFU per larva was determined over 18 days (Figure 5(a)). On observation, the infected larvae showed more death events from the 12^th^ day postinfection. In comparison between the strains, lysX mutant was highly virulent for *Galleria*, because within 18 days all the animals infected with the lysX mutant were killed. After 18 days following infection only 40% mortality was recorded in the larvae group infected with the wild-type MAH strain, whereas mortality after infection with the lysX mutant was 100%. In parallel, the lysX complemented strain showed an intermittent behavior between wild type and the mutant with 65% mortality. While more than 50% of larvae survival was recorded for the wildtype (undefined median survival time), the mutant displayed a median survival time of 13 days and lysX complemented strain of 15 days, respectively. Conclusively, the lysX mutant strain revealed to be more virulent than the wild type with respect to the overall mortality of *G. mellonella larvae* (Figure 5(a)). The survival of MAH (infection dose 10^6^ CFU) within *G. mellonella* was determined over a period of 10 days postinfection. After infection of *G. mellonella* larvae with the MAH strains, an approximate 50% decrease in the bacterial numbers was observed within 120 h. Anyhow, the bacterial count *in-vivo* was elevated in all the three strains between 120 h and 240 h postinfection. However, this increase was most pronounced in the case of infection by the lysX mutant. While the bacterial numbers upon infection with the wild-type strain increased by a factor of around tenfold during this time period, the bacterial numbers for the lysX mutant increased around 100 fold (Figure 5(b)). The lysX mutant thus displayed higher replication rates within *G. mellonella* larvae than the wild type.

## Discussion

The *lysX* gene has been hypothesized to be correlated with intracellular reproductivity and as an important variable for the modulation of virulence in *M. tuberculosis* [40]. This was postulated based on a previous study which reported that the lysX mutant from MTB exhibited a growth defect in THP-1 macrophages and in mouse lungs and was found to be attenuated in guinea pig’s lungs [26]. It was also demonstrated that a higher expression of *lysX* in MTB strains was correlated with increased levels of intracellular survival in both *in vivo* and *in vitro* and also inducing more severe lesions related with pneumonia [40], thus indicating *lysX* as a necessary factor for acquiring full virulence.

Intriguingly, our studies on the *lysX* gene from MAH revealed a different phenotype both *in vivo* and *in vitro*. Bioinformatics comparison of the LysX protein sequence of different mycobacteria and of the nucleic-acid sequences of an extended lysX gene region revealed differences both among different mycobacterial species as well as among different strains from the same species. As shown in supplementary Table S1 and S2 the length of the LysX protein varies, mainly caused by different length of the 5ʹ region of the gene. The amino acid identity to the LysX protein from MAH 104 was 99% to MAH TH135 and 91% to MAH k10. The phylogenetically more distant species *M. tuberculosis* and *M. leprae* displayed identities of 77% and 82%, respectively, to the MAH 104 LysX protein. Also, blast analysis with the region containing the *lys*X** gene from MAH 104 and with other MAH and *M. tuberculosis* strains demonstrated differences in gene sequence and genome organization especially in the *M. tuberculosis* strains (Fig. S1). These differences might have contributed to the diverging phenotypic effects of *lysX* mutations in MAH and *M. tuberculosis*.

Phenotypic traits detected in *M. avium* indicate that in contrast with those described in MTB, *M. avium* adopts distinct strategies to manipulate the host immune response and persist intracellularly. In this context, we focused on studying the interference of the MAH *lysX* gene on the host-pathogen interplay and its contribution to the differential virulence features.

The host mounts a complex response when infected with mycobacteria to kill them or to limit their proliferation. Reactive oxygen and nitrogen intermediates (RONS) have been demonstrated as major antimicrobial molecules produced by the human host during the course of infection with MTB [41]. Under extracellular growth conditions, the lysX mutant from MAH proved to be slightly more sensitive toward H_2_O_2_ and DETA-NO. This behavior was in accordance with our previous proteomics studies which showed that some of the genes which are implicated in resistance toward RONS were downregulated in the lysX mutant in comparison to the wild type and complemented strains [28]. These include the genes encoding catalase (*katG*) [42], alkyl hydroperoxide reductase (*ahpC*) and thioesterase family protein (*MAV_2246*). The ROS levels induced by the host immune cells also determine the ability of MTB to survive and replicate in the host [43]. However, this was not the case in *Mycobacterium avium* complex (MAC) infections, wherein it was shown that there was no significant relationship between the degree of resistance to the host effector molecules (RONS, free fatty acids (FFA)) and its virulence in mice [44].

MAH is a pathogen that establishes infection by crossing the respiratory and intestinal mucosas in both humans and other mammals [27]. High concentrations of antimicrobial peptides (AMPs) are encountered by the bacterium in these mucus layers [45]. The AMPs are an important component in host innate response against bacteria and they are also released intracellularly in phagocytic cells as in macrophages and neutrophils [27]. The human beta defensin HBD-1 plays a role in the innate immune defense against MTB by permeabilisation of both the mycobacterial cell wall and cell membrane [46]. Furthermore, significant levels of β-defensins have been detected in bronchoalveolar lavage fluid from patients with *M. avium* infection [47]. We, therefore, attempted to assess the impact of HBD-1 on the survival of MAH depending on the presence of *lysX*. *In-vitro* testing with the *M. avium* strains in response to HBD-1 showed a decrease in the viability of the lysX mutant. This result goes well with findings about the effect of MrpF (corresponding to the 5ʹend domain from LysX of MAH) from *Staphylococcus aureus*. It was proven that staphylococcal *mprF* confers resistance toward AMPs including defensins [46]. MprF is a unique enzyme which converts the anionic phospholipids to cationic lipids in the bacterial membrane with l‐lysine or l‐alanine, thus changing the membrane surface charge and shrinking the affinity for cationic defensins [48]. Maloney and colleagues have also demonstrated that in the absence of the *lysX* gene, MTB becomes susceptible to AMPs *in vitro* [26]. Cytokines are of vital importance in the case of mycobacterial infections as they serve as effectors and regulators of innate immune defense, also determining the consequential adaptive T cell response, yet their regulatory mechanism and their role in mycobacterial infections are still poorly understood [49]. The induction of cytokine secretions are also driven by *M. avium* morphotype and virulence [50]. Our evaluation of selected cytokine expression by freshly isolated PBMCs infected with the MAH strains revealed that the lysX mutant strain induced higher cytokine levels (IL-1β, IL- 12, TNFα and IL-10) than the wild type and complemented strains. MTB mutants which lack the production of some cell-wall components (eg. phenolic glycolipid (PGL), phthiocerol dimycocerosate (PDIM)) also induce an increased secretion of the proinflammatory cytokines TNF-α, IL-6, and IL-12 compared to the wild type [51]. The increased secretion of inflammatory cytokines by the lysX mutant could be partly explained by the fact that LysX already plays a role in the production of membrane–bound lysylphosphatidylglycerol. Moreover, using proteomic mass spectrometry, we identified genes involved in glycerophospholipid metabolism (*MAV_1825, fbpB*, *fbpC*) and GPL synthesis (*MAV_4518, rmt4/mftb, fadE5*, *sap*) that were differentially regulated in the lysX mutant when compared to the wild type and lysX complemented strains.

Highly stimulated cells of macrophage lineage form MGCs at a terminal stage of differentiation. MGC are generally produced by cell–cell fusion [52,53] and it was recently reported that the fusion process might be triggered in a TLR2-dependent cell activation through lipomannan from mycobacteria [54].

Upon the infection of IFNγ-activated HBDM with the *M. avium* strains, the lysX mutant induced a higher rate of macrophage fusion than the wild type and the complemented strain. In correspondence, the gene *DprE1* which catalyzes the synthesis of cell-wall arabinans was strongly overexpressed in the lysX mutant. Besides the DprE1 was also illustrated to be essential for the growth and survival of MTB [55].

It is evident from our experiments that lysX mutation in MAH has an impact on several virulence attributes. Further confirmation of a role of LysX for virulence was obtained by performing *in-vivo* infection experiments using the invertebrate infection model *G. mellonella. G. mellonella* has a sophisticated innate immune system which consists of cellular and humoral defenses, which expose of a functional similarity to vertebrates [56]. Recent reports have already demonstrated the potentiality of employing a wax moth larvae model for studying both slow and fast-growing mycobacteria infections such as *M. bovis BCG* [57], *M. abscessus* [58], *M. fortuitum* [59], *M. marinum* [59] and *M. aurum* [59].

In this study we have demonstrated the suitability of *G. mellonella* as an infection model for MAH and also employed this *in-vivo* model to assess the virulence of our strains. The survival of both the host and mycobacteria was determined over a period after infection of 18 days or 10 days, respectively (Figure 5). Though the strains did not show the same pattern of dying in all the experimental trials, on comparison using the log-rank test, the survival curves between wild type and mutant were found to be significantly different (p < 0.0001). The lysX mutant strain was highly virulent, killing 100% of larvae within a few days. In addition, the mutated strain also featured a significant growth *in-vivo* between 5 and 10 days after infection, thus indicating a persistent viable mycobacterial infection compared to the wild type and complemented strains, which showed only very moderate growth. Ten days after infection the mutant had multiplied more than 100 fold and all the infected larvae were dead at the end of the experiment. Interestingly, the *in-vivo* growth pattern of the MAH lysX mutant and wild type in *Galleria* were also comparable to the growth pattern observed *in-vitro* in HBDM in so far as the mutant showed increased growth at the later time points of infection [28]. This phenotype is coherent with our proteomics data which hitherto revealed that the LysX deficiency in MAH arouses a metabolic alteration which represents an intracellular metabolic status found in MTB, thereby equipping the bacteria for intracellular niches of the host organism [28]. In particular, the gene class of ESX export systems which play a pivotal role in mycobacterial virulence [60] (*EccA3*, *MAV_4606, MAV_0940*) and those involved in anti-apoptotic pathways (*CysK* [61], *KatG* [62], *NuoG* [63]) were also differentially regulated in the lysXmut strain.

The lysX mutant from MTB exposed an opposite phenotype by displaying a growth defect in mouse and guinea pig lungs and reduced pathology relative to wild type [26]. Glycopeptidolipids (GPLs) are major cell wall constituents of *M. avium* and other NTM species but MTB and *M. leprae* are devoid of them [64]. It has been hypothesized that the GPLs play important roles in MAC infections [24] and are involved in immunomodulatory activities by influencing cytokine induction [64] and promoting phagocytosis by inhibiting phagosome-lysosome fusion. Recent studies report that the macrophages infected with *M. avium* expressing a deficient GPL expression produce more cytokines and inflammatory chemokines like TNF-α, IL-6, IL-12p40, and RANTES [65]. Thus, the elevated inflammatory response induced by the lysX mutant strain may also be linked to the GPL expression pattern.

Differential expression of GPLs on the surface of mycobacteria might also be a phenotypic feature accounting for the different effects on host immune responsiveness as they possess the ability to interact with cell membranes [66,67]. Likewise, variations in GPL structure affect the pathogenicity of MAC organism [24].

In summary, the *lysX* gene from MAH was observed to play a role in the modulation of mycobacterial virulence which contributes knowledge toward the pathophysiology of MAH and its interaction with the host. The study also sheds light on fundamental differences between MTB and *M. avium* in terms of their survival strategies.
