# Supplementary material for: Mutation on lysX from Mycobacterium avium hominissuis impacts the host–pathogen interaction and virulence phenotype
Source: Virulence. 2020 Jan 29;11(1):132–44. doi: 10.1080/21505594.2020.1713690 (PMC6999840; doi:10.1080/21505594.2020.1713690)
Supplement: Supplemental Material [file kvir-11-01-1713690-s001.zip › Supplementary Fig caption.docx]

**Supplementary Fig. S1**

ClustalW Alignment performed with Geneious (Bio-informatics software) of the genomic region carrying the lysX gene in MAH 104 plus three genes located upstream (rplT, rpmI, infC) and three genes located downstream (putative esterase family protein gene, tetR family protein gene, ThiF family protein gene) from lysX from strains M. avium subsp. hominissuis (MAH) 104 (accession CP000479), MAH H87 (accession CP018363), MAH mc2 2500 (accession CP036220), MAH OCU901s S2 2s (accession CF018014), MAH TH135 (accession AP012555), MAH HP17 (accession CP016818), M. tuberculosis (MTB) H37Rv (accession CP003248) and MTB Haarlem (accession CP00164).
