# Supplementary material for: Mutation on lysX from Mycobacterium avium hominissuis impacts the host–pathogen interaction and virulence phenotype
Source: Virulence. 2020 Jan 29;11(1):132–44. doi: 10.1080/21505594.2020.1713690 (PMC6999840; doi:10.1080/21505594.2020.1713690)
Supplement: Supplemental Material [file kvir-11-01-1713690-s001.zip › Table S1_revision_Kirubakar_Lewin.docx]

**Supplementary Table xx: Comparison of LyX proteins from *M. avium* subsp. *homininssuis,* *M. avium* subsp. *paratuberculosis*, *M. tuberculosis* and *M. leprae***

| **(sub)species, strain,** | **accession** | **length of LysX [aa]** | **% identity [aa] with LysX from MAH 104^*^** |
| --- | --- | --- | --- |
| MAH, 104 | CP000479 | 1,075 | 100 |
| MAH, TH135 | AP012555 | 1,085 | 99 |
| MAP, k10 | AE016958 | 1,177 | 91 |
| *M. tuberculosis*, H37Rv | NC_000962 | 1,172 | 77 |
| *M. lepra*, TN | NC_002677 | 1,039 | 82 |

^*^calculated with Geneious alignment (Global Alignment);
MAH: *M. avium* subsp. *homininssuis*; MAP: *M. avium* subsp. *paratuberculosis*; aa: amino acids
