# Supplementary figures and images for: Mutation on lysX from Mycobacterium avium hominissuis impacts the host–pathogen interaction and virulence phenotype
Source: Virulence. 2020 Jan 29;11(1):132–44. doi: 10.1080/21505594.2020.1713690 (PMC6999840; doi:10.1080/21505594.2020.1713690)

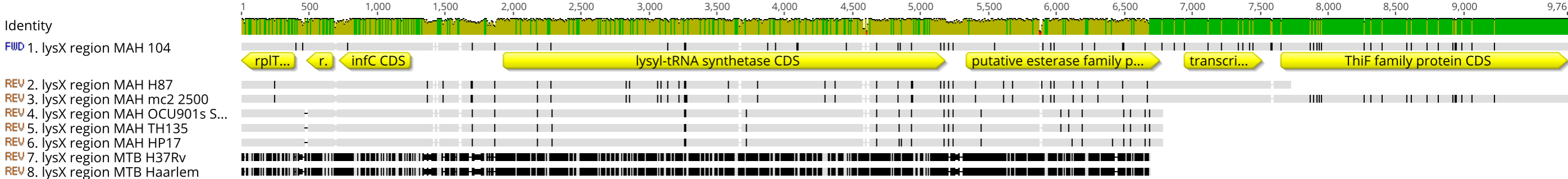

Supplement: Supplemental Material [file kvir-11-01-1713690-s001.zip › Fig. S1_revision_Kirubakar_Lewin.pdf]

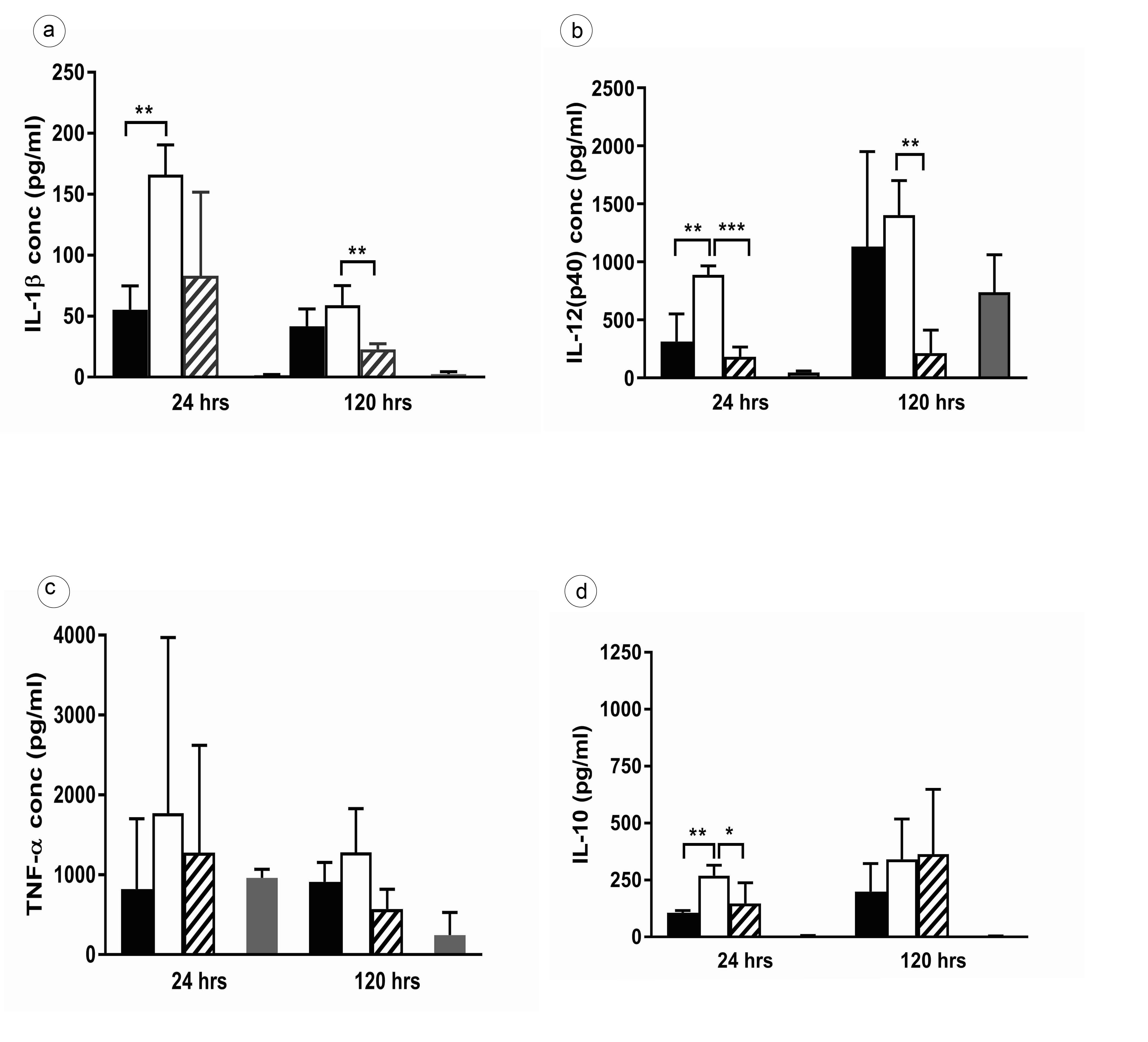

Supplement: Supplemental Material [file kvir-11-01-1713690-s001.zip › Fig2_revision_Kirubakar_Lewin.jpg]
