# Supplementary material for: Mutation on lysX from Mycobacterium avium hominissuis impacts the host–pathogen interaction and virulence phenotype
Source: Virulence. 2020 Jan 29;11(1):132–44. doi: 10.1080/21505594.2020.1713690 (PMC6999840; doi:10.1080/21505594.2020.1713690)
Supplement: Supplemental Material [file Table S2_revision_Kirubakar_Lewin.pdf › Table S2_revision_Kirubakar_Lewin.pdf]

Supplementary Table S2: Alignment of the LysX proteins from *M. avium* subsp. *hominissuis* (MAH) strains 104 and TH135, *M. avium* subsp. *paratuberculosis* (MAP) strain k10, *M. tuberculosis* strain H37Rv and *M. leprae* strain TN.
For accession numbers see Supplementary Table S1.

LysX MAH 104	- -
LysX MAH TH135	- -
LysX MAP k10	M R R A G R S R Q F S S V E E A F S T S A A R P G P R G R R S G R E N T A K F V P A T L S A S T S F S R I E G I S S R S V T L A S P G S R S G S G P R S G P R L
LysX Mtb H37Rv	- - - - - - - - - - - - M G L H L T V P G L R R D G R G V Q S N S H D T S S K T T A D I S R C P Q H T D A G - L Q R A A T P G I S R L L G I S S R S V T L T K P
LysX Ml TN	- -

LysX MAH 104	- M I A T V S L L G S V S P L I R Y L I K V P R E F I N D Y L F N F P D T S I A W S F V L A L L A A A L T A R K R I A
LysX MAH TH135	- - - - - - - - - - - - M P A A A G W T V G V I A T V S L L G S V S P L I R Y L I K V P R E F I N D Y L F N F P D T S I A W S F V L A L L A A A L T A R K R I A
LysX MAP k10	G P R N R S T S R Y R W V P A A A G W T V G V I A T V S L L G S V S P L I R Y L I K V P R E F I N D Y L F N F P D T S I A W S F V L A L L A A A L T A R K R I A
LysX Mtb H37Rv	R S A T R G N S R Y H W V P A A A G W T V G V I A T L S L L A S V S P L I R W I I K V P R E F I N D Y L F N F P D T N F A W S F V L A L L A A A L T A R K R I A
LysX Ml TN	- M L A L L A A A L T T R K R I A

LysX MAH 104	W L L L L G N M I L A A A L N V A D I A A G D N T A A E I F G E N L G F A V H I V A I V L L V L A Y R E F W A K V R K G A L V K A A A V L V A G D V V G I L V S
LysX MAH TH135	W L L L L G N M I L A A A L N V A D I A A G D N T A A E I F G E N L G F A V H I V A I V L L V L A Y R E F W A K V R K G A L V K A A A V L V A G D V V G I L V S
LysX MAP k10	W L L L L G N M I L A A A L N V A D I A A G D N T A A E I F G E N L G F A V H I V A I V L L V L A Y R E F W A K V R K G A L V K A A A V L V A G D V V G I L V S
LysX Mtb H37Rv	W L V L L A N M V L A A V V N A A E I A A G G N T A A E S F G E N L G F A V H V V A I V V L V L G Y R E F W A K V R R G A L F R A A A V W L A G A V V G I V A S
LysX Ml TN	W L L L L G N M V L A A V L N A V D M A A N G N T P A E T F G E N L G F A V H V V A I L L L V L S Y R E F W A K V R R G A L F K A A A V L V A G D V I G I L L S

LysX MAH 104	W G L V E L F P G T L A R Q D R L P Y V V N R V V G F A L A D P D L F T G R P H V F L N A I F G L F G A L A L I M A T I V L F Q S Q R A E N A L T G E D E S A I
LysX MAH TH135	W G L V E L F P G T L A R Q D R L P Y V V N R V V G F A L A D P D L F T G R P H V F L N A I F G L F G A L A L I M A T I V L F Q S Q R A E N A L T G E D E S A I
LysX MAP k10	W G L V E L F P G T L A R Q D R L P Y V V N R V V G F A L A D P D L F T G R P H V F L N A I F G L F G A L A L I M A T I V L F Q S Q R A E N A L T G E D E S A I
LysX Mtb H37Rv	W G L V E L F P G S L A P D E R L G Y A A N R V V G F A L A D P D L F T G R P H V F L N A I F G L F G A F A L I G A A I V L F L S Q R A D N A L T G E D E S A I
LysX Ml TN	L G L V E L F P G S L A R Q D R L P Y V A N R V V G F A L A D P D L F S G K P H V L L N A I F G L F G A L A L I M A T I V L F Q S Q R A D N A L T G E D E S A I

LysX MAH 104	R G L L E L Y G K N D S L G Y F A T R R D K S V V F A Q S G R A A I T Y R V E I G V C L A S G D P I G D P R A W P Q A V D A W L G L C Q T Y G W A P G V M G A S
LysX MAH TH135	R G L L E L Y G K N D S L G Y F A T R R D K S V V F A Q S G R A A I T Y R V E I G V C L A S G D P I G D P R A W P Q A V D A W L G L C Q T Y G W A P G V M G A S
LysX MAP k10	R G L L E L Y G K N D S L G Y F A T R R D K S V V F A Q S G R A A I T Y R V E I G V C L A S G D P I G D P R A W P Q A V D A W L G L C Q T Y G W A P G V M G A S
LysX Mtb H37Rv	R G L L D L Y G K D D S L G Y F A T R R D K S V V F A S S G R A C I T Y R V E V G V C L A S G D P V G D H R A W P Q A V D A W L R L C Q T Y G W A P G V M G A S
LysX Ml TN	R G L L E L Y G K N D S L G Y F A T R R D K S V I F A H N G R A A I T Y R V E I G V C L A S G D P V G D P G A W P Q A V D A W L E L C Q T Y G W A P G V M G A S

LysX MAH 104	T Q G A R T Y R E A G L N A L E L G D E A I L R T S E F K L S G P D M R G V R Q A V T R A R R A G L T V R I R R H R D I S P E A M A D T I A R A D A W R D T Q T
LysX MAH TH135	T Q G A R T Y R E A G L N A L E L G D E A I L R T S E F K L S G P D M R G V R Q A V T R A R R A G L T V R I R R H R D I S P E A M A D T I A R A D A W R D T Q T
LysX MAP k10	T Q G A R T Y R E A G L N A L E L G D E A I L R T S E F K L S G P D M R G V R Q A V T R A R R A G L T V R I R R H R D I S P E A M A D T I A R A D A W R D T Q T
LysX Mtb H37Rv	S Q G A Q T Y R E A G L T A L E L G D E A I L R P A D F K L S G P E M R G V R Q A V T R A R R A G L T V R I R R H R D I A E D E M A Q T I T R A D S W R D T E T
LysX Ml TN	S Q G A Q V F R R A G F N A I E L G D E A I L R T A V Y K L S G P D M R G V R Q A V T R A R R A G L T V R I R R H S D I S A N E M A D T I A R A D A W R D T E F

LysX MAH 104	E R G F S M A L G R L G D P A D G D C L L V E A I D R D G S V V A M L S L V P W G T T G V S L D L M R R S P S S P N G T I E L M V S E L A L N A E S L G I T R I
LysX MAH TH135	E R G F S M A L G R L G D P A D G D C L L V E A I D R D G S V V A M L S L V P W G T T G V S L D L M R R S P S S P N G T I E L M V S E L A L N A E S L G I T R I
LysX MAP k10	E R G F S M A L G R L G D P A D G D C L L V E A I D R D G S V V A M L S L V P W G T T G V S L D L M R R S P S S P N G T I E L M V S E L A L N A E S L G I T R I
LysX Mtb H37Rv	E R G F S M A L G R L G D P A D S D C L L V E A I D P H N Q V L A M L S L V P W G T T G V S L D L M R R S P Q S P N G T I E L M V S E L A L H A E S L G I T R I
LysX Ml TN	E R G F S M A L G R L G D P A D S D C L L V E A V D R D D H V V A I L S L V P W G T T G V S L D V M R R S P Q S P N G T I E L M V S E L A L K G E T L G I A R I

LysX MAH 104	S L N F A M F R S A F E Q G A Q L G A G P V A R L W R G L L L F F S R W W Q L E T L Y R S N M K Y Q P D W V P R Y A C Y E D A R L I P R V G V A S V I A E G F L
LysX MAH TH135	S L N F A M F R S A F E Q G A Q L G A G P V A R L W R G L L L F F S R W W Q L E T L Y R S N M K Y Q P D W V P R Y A C Y E D A R L I P R V G V A S V I A E G F L
LysX MAP k10	S L N F A M F R S A F E Q G A Q L G A G P V A R L W R G L L L F F S R W W Q L E T L Y R S N M K Y Q P D W V P R Y A C Y E D A R L I P R V G V A S V I A E G F L
LysX Mtb H37Rv	S L N F A V F R A A F E Q G A Q L G A G P V A R L W R G L L V F F S R W W Q L E T L Y R S N M K Y Q P E W V P R Y A C Y E D A R V I P R V G V A S V I A E G F L
LysX Ml TN	S L N F T M F R A A F E Q G A Q L G A G P I A R L W R G L L L F F S R W W Q L E T L Y R S N I K Y L P D W V P R Y A C Y E D A R L I P R V G V A S V I A E G F L

LysX MAH 104	V L P F S R R D K V H T G H H P A V P A R L A Q S G L L H H D G S A P D V S G L R P E - R T D A E E A R S R L P E Q V R V R L A K L K V L Q R N G V D A Y P V G
LysX MAH TH135	V L P F S R R D K V H T G H H P A V P A R L A Q S G L L H H D G S A P D V S G L R P E - R T D A E E A R S R L P E Q V R V R L A K L K V L Q R N G V D A Y P V G
LysX MAP k10	V L P F S R R D K V H T G H H P A V P A R L A Q S G L L H H D G S A P D V S G L R P E - R T D A E E A R S R L P E Q V R V R L A K L K V L Q R N G V D A Y P V G
LysX Mtb H37Rv	V L P F S R R N R V H T G H H P A V P E R L A A T G L L H H D G S A P D V S G L R Q V G L T N G D G V E R R L P E Q V R V R F D K L E K L R S S G I D A F P V G
LysX Ml TN	V L P F S R R G R V H T G H H P A V P A R L A E S G L L H H D G S T P D V S G L Q T A - D V D L E V A N S R I P E Q V R V R L A K L K T L Q L N G I D A Y P V G

LysX MAH 104	C P P S H T V A Q A L D A D D Q Q D I T V A G R I L R I R D F G G V L F A Q L R D W S G E M Q V L L D N S R L E R G R T A D F T A A I D L G D L V E V S G Q M G
LysX MAH TH135	C P P S H T V A Q A L D A D D Q Q D I T V A G R I L R I R D F G G V L F A Q L R D W S G E M Q V L L D N S R L E R G R T A D F T A A I D L G D L V E V S G Q M G
LysX MAP k10	C P P S H T V A Q A L D A D D Q Q D I T V A G R I L R I R D F G G V L F A Q L R D W S G E M Q V L L D N S R L E R G R T A D F T A A I D L G D L V E V S G Q M G
LysX Mtb H37Rv	R P P S H T V A Q A L A A D H Q A S V S V S G R I M R I R N Y G G V L F A Q L R D W S G E M Q V L L D N S R L D Q G C A A D F N A A T D L G D L V E M T G H M G
LysX Ml TN	H P P S H T V A Q A L D A D D E G T V S I S G R I L R I R D Y G G V L F A H V R D W S G E I Q V L L D N L V L E C C C I A D F T A A I D L G D I V E M T G N M G

LysX MAH 104	F S K K G T R S L I V T D W R M I G K C L R P L P N K W K G L T D P E A R V R T R Y V D L A V N P E S R E L I A A R S E V L R S V R Q T L F A K G F I E V E T P
LysX MAH TH135	F S K K G T R S L I V T D W R M I G K C L R P L P N K W K G L T D P E A R V R T R Y V D L A V N P E S R E L I A A R S E V L R S V R Q T L F A K G F I E V E T P
LysX MAP k10	F S K K G T R S L I V T D W R M I G K C L R P L P N K W K G L T D P E A R V R T R Y V D L A V N P E S R E L I A A R S E V L R S V R Q T L F A K G F I E V E T P
LysX Mtb H37Rv	A S K T G T P S L I V S G W R L I G K C L R P L P N K W K G L L D P E A R V R T R Y L D L A V N A E S R A L I T A R S S V L R A V R E T L F A K G F V E V E T P
LysX Ml TN	F S K N G T R S L I V R N W R L I G K C L R P L P N K W K G L T D P E A R V R T R Y V D L A V N T E S R N L I M A R S C V L R S V R E M L F A K G F V E V E T P

LysX MAH 104	I L Q Q I H G G A T A R P F V T H I N T Y D M D L F L R I A P E L Y L K R L C V G G V E R V F E L G R A F R N E G V D F S H N P E F T L L E A Y Q A H A D Y R V
LysX MAH TH135	I L Q Q I H G G A T A R P F V T H I N T Y D M D L F L R I A P E L Y L K R L C V G G V E R V F E L G R A F R N E G V D F S H N P E F T L L E A Y Q A H A D Y R V
LysX MAP k10	I L Q Q I H G G A T A R P F V T H I N T Y D M D L F L R I A P E L Y L K R L C V G G V E R V F E L G R A F R N E G V D F S H N P E F T L L E A Y Q A H A D Y R V
LysX Mtb H37Rv	I L Q Q L H G G A T A R P F V T H I N T Y S M D L F L R I A P E L Y L K R L C V G G V E R V F E L G R A F R N E G V D F S H N P E F T L L E A Y Q A H A D Y L E
LysX Ml TN	I L Q Q I H G G A T A R P F A T R I N T Y D M D L F L R I A P E L Y L K R L C I G G V E R V F E L G R A F R N E G V D F S H N P E F T L L E A Y Q A H A D Y L M

LysX MAH 104	W I D G C R E L I Q N A A Q A A H G E Q T V L R P - - - - G A D G - - - R L Q P V D I S G I W A V K T V H D A V S E A L G E Q V D P G T S L S T L R K L S D A A
LysX MAH TH135	W I D G C R E L I Q N A A Q A A H G E Q T V L R P - - - - G A D G - - - R L Q P V D I S G I W A V K T V H D A V S E A L G E Q V D P G T S L S T L R K L S D A A
LysX MAP k10	W I D G C R E L I Q N A A Q A A H G E Q T V L R P - - - - G A D G - - - R L Q P V D I S G I W A V K T V H D A V S E A L G E Q V D P G T S L S T L R K L S D A A
LysX Mtb H37Rv	W I D G C R E L I Q N A A Q A A N G A P I A M R P R T D K G S D G T R H H L E P V D I S G I W P V R T V H D A I S E A L G E R I D A D T G L T T L R K L C D A A
LysX Ml TN	W I D G C R E L I Q N A A E A A N G T Q T L M R P R I E - G A S G T A N H L E P I D I S G V W P V K T V Y E A V S E A L G E C V D T S T E L A T L R K L S D A A

LysX MAH 104	R I P Y R A H W D A G A V V L E L Y E H L V E D R T E E P T F Y V D F P T S V S P L T R P H R S R P G V A E R W D L V A W G V E L A T A Y S E L T D P V E Q R R
LysX MAH TH135	R I P Y R A H W D A G A V V L E L Y E H L V E D R T E E P T F Y V D F P T S V S P L T R P H R S R P G V A E R W D L V A W G V E L A T A Y S E L T D P V E Q R R
LysX MAP k10	R I P Y R A H W D A G A V V L E L Y E H L V E D R T E E P T F Y V D F P T S V S P L T R P H R S R P G V A E R W D L V A W G V E L A T A Y S E L T D P V E Q R R
LysX Mtb H37Rv	G V P Y R T Q W D A G A V V L E L Y E H L V E C R T E Q P T F Y I D F P T S V S P L T R P H R S K R G V A E R W D L V A W G V E L G T A Y S E L T D P V E Q R R
LysX Ml TN	H I P Y W P H W D T G A V V L K L Y E H L V E N R T D Q P T F Y I D F P T S V S P L T R P H R S K P G V A E R W D L V A W G I E L G T A Y S E L T D P V E Q R R

LysX MAH 104	R L Q E Q S L L A A G G D P E A M E L D E D F L Q A M E Y A M P P T G G L G M G V D R L V M L I T G R S I R E T L P F P L A K P H
LysX MAH TH135	R L Q E Q S L L A A G G D P E A M E L D E D F L Q A M E Y A M P P T G G L G M G V D R L V M L I T G R S I R E T L P F P L A K P H
LysX MAP k10	R L Q E Q S L L A A G G D P E A M E L D E D F L Q A M E Y A M P P T G G L G M G V D R L V M L I T G R S I R E T L P F P L A K P H
LysX Mtb H37Rv	R L Q E Q S L L A A G G D P E A M E L D E D F L Q A M E Y A M P P T G G L G M G I D R V V M L I T G R S I R E T L P F P L A K P H
LysX Ml TN	R L H E Q S L L A V G G N P E A M E L D E D F L Q A M E Y A M P P T G G L G M G I D R L V M L I T G R S I R E T L P F P L A K P H
